# Histiocytoid Sweet's syndrome presenting with annular erythematous plaques*

**DOI:** 10.1590/abd1806-4841.20164361

**Published:** 2016

**Authors:** Renata Marcarini, Raquel Nardelli de Araujo, Monisa Martins Nóbrega, Karina Bittencourt Medeiros, Alexandre Carlos Gripp, Juan Manuel Piñeiro Maceira

**Affiliations:** 1 Universidade do Estado do Rio de Janeiro (UERJ) – Rio de Janeiro (RJ), Brazil; 2 Universidade Federal do Espírito Santo (UFES) – Vitória (ES), Brazil; 3 Universidade Federal do Rio de Janeiro (UFRJ) – Rio de Janeiro (RJ), Brazil

**Keywords:** Pharyngitis, Sweet's Syndrome, Skin and connective tissue diseases

## Abstract

Histiocytoid Sweet's Syndrome is a rare inflammatory disease described in 2005 as a variant of the classical Sweet's Syndrome (SS). Histopathologically, the dermal inflammatory infiltrate is composed mainly of mononuclear cells that have a histiocytic appearance and represent immature myeloid cells. We describe a case of Histiocytoid Sweet's Syndrome in an 18-year-old man. Although this patient had clinical manifestations compatible with SS, the cutaneous lesions consisted of erythematous annular plaques, which are not typical for this entity and have not been described in histiocytic form so far. The histiocytic subtype was confirmed by histopathological analysis that showed positivity for myeloperoxidase in multiple cells with histiocytic appearance.

## INTRODUCTION

Sweet's syndrome (SS) is characterized by a variety of symptoms and histological findings that include skin lesions, systemic manifestations of acute disease and a dermal inflammatory infiltrate with a significant neutrophilic component. In general, SS rapidly responds to systemic corticosteroid treatment. The disease can be divided into three groups: classical, malignancy-associated and drug-induced.^1^

The classical form may be associated with infections, inflammatory bowel disease or pregnancy.^1^ Malignancy-Associates Sweet's Syndrome is most related to acute myeloid leukemia.^2^ The drug-induced variant is usually related to granulocyte-colony stimulating factor.^3^ The diagnosis is based on major and minor criteria by recognizing the clinical, laboratory and histopathological findings, as well as excluding other diseases that may have similar characteristics (Chart 1).^4^

**Chart 1:** Diagnostic criteria for classical SS

1. Abrupt onset of painful erythematous plaques or nodules
2. Histopathologic evidence of a dense neutrophilic infiltrate without evidence of leukocytoclastic vasculitis
3. Pyrexia >38°C
4. Association with an underlying hematologic or visceral malignancy, inflammatory disease, or pregnancy, or precedent for an upper respiratory or gastrointestinal infection or vaccination
5. Excellent response to systemic corticosteroids or potassium iodide
6. Abnormal laboratory values at presentation (three out of four): erythrocyte sedimentation rate >20 mm/hr; positive C-reactive protein; >8,000 leukocytes; >70 % neutrophils
a) The presence of both major criteria (1 and 2), and two of the four minor ones are essential for the diagnosis of classical SS

## CASE REPORT

An 18-year-old male patient presented with nonsuppurative tonsillitis for which he made use of dipyrone, ibuprofen and ciprofloxacin. Seven days later, oropharyngeal pain was relieved, but the patient reported high fever, chills, polyarthralgia, macroscopic haematuria and hyperemia accompanied by burning sensation in his eyes. Concomitantly, two papular, well-defined, non-pruritic and painless lesions appeared in the right temporal region.

**Figure 1:**
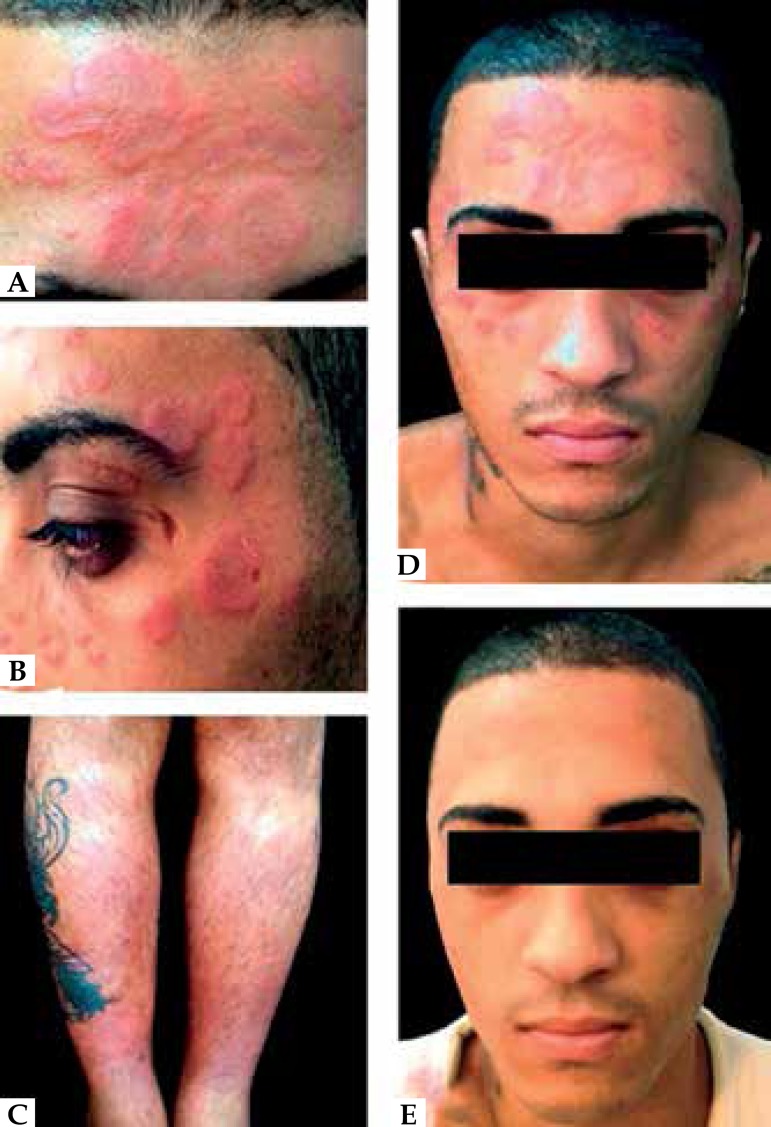
Cutaneous manifestations. **A)** Annular lesions on the forehead; **B)** Annular lesions on the temporal region; **C)** Infiltrative erythematous lesions on the lower limbs; **D)** Annular lesions on the face; **E)** Satisfactory response to corticosteroids (second day)

After five days, the patient presented with arthritis in the knees and ankles, along with daily fever. The lesions increased in number and size, progressing to erythematous plaques with raised and sharply demarcated borders, central clearing, forming an annular pattern without a pseudovesicular appearance (Figure 1). We also noted splenomegaly and edema of the legs, accompanied by infiltrative erythematous lesions and palpable tender nodules on the pretibial regions (Figure 1).

Laboratory tests showed leukocytosis (13,830 leukocytes/ mm^3^) with neutrophilia (84% neutrophils), erythrocyte sedimentation rate of 65mm/h and ferritin of 419µg. Serology for Epstein-Barr, cytomegalovirus, toxoplasma, human immunodeficiency virus (ELISA and quantitative PCR), syphilis (VDRL and TPHA), hepatitis B and C and antistreptolysin antibody were negative. CAT scans of the chest, abdomen and pelvis only revealed splenomegaly. Antinuclear factor, antineutrophil cytoplasmic antibodies and rheumatoid factor were also negative. The analysis of the peripheral blood smear showed no abnormalities.

Based on the clinical findings, we suspected SS. Methylprednisolone 62.5 mg/day was prescribed (equivalent to 1mg/kg of prednisone). After two days, we noted significant clinical and laboratory improvements (Figure 1).

**Figure 2:**
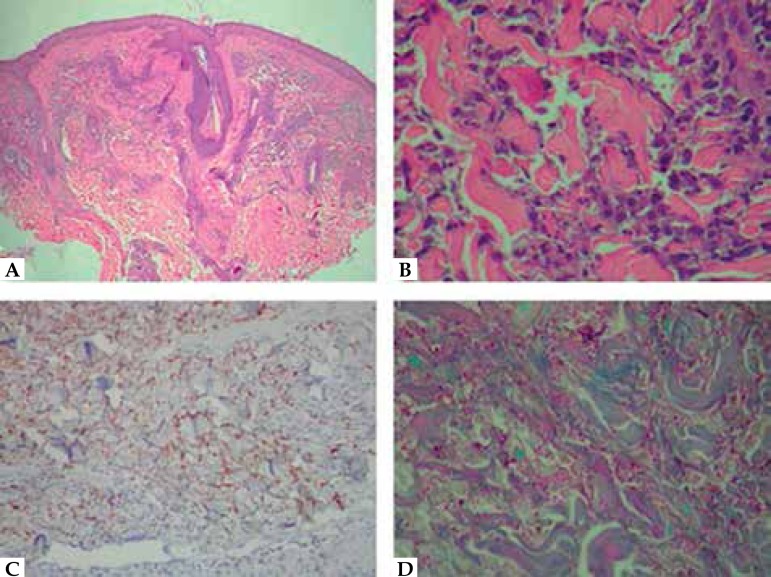
Skin Biopsy. **A, B)** Diffuse inflammatory infiltrate of mononuclear cells in the upper dermis with a predominance of histiocytes without edema (hematoxylin-eosin, A x40, B x400); **C)** immunostaining with myeloperoxidase in numerous cells of the dermis (avidin-biotin technique, x100); **D)** dermal interstitial infiltrate of histiocytic appearance cells with granular cytoplasm (PAS, x400)

Biopsy of the temporal lesion revealed a diffuse inflammatory infiltrate of mononuclear cells in the upper dermis without edema. Many of these cells resembled histiocytes with a cytoplasmic granular appearance, in a perivascular and interstitial distribution. We identified few neutrophils, eosinophils and sparse leukocytoclasia (Figure 2). The epidermis was spared and there were no signs of vasculitis. The dermo-epidermal junction showed discrete vacuolar degeneration. Periodic acid-Schiff and Grocott staining methods revealed no microorganisms. Immunostaining detected myeloperoxidase activity in numerous cells of histiocytic appearance (Figure 2), suggesting Histiocytoid Sweet's syndrome (HSS).

## DISCUSSION

SS is considered a neutrophilic dermatosis with a not well established pathogenesis. The case reported meets the diagnostic criteria of SS, such as laboratory abnormalities, acute onset of lesions, fever and excellent clinical response to corticosteroid. However, it was not possible to define the etiology of the SS described above since the patient made use of three medications together and also had a previous oropharyngeal infection, both possible causes described for SS.

The typicall lesions of SS are presented with edematous, non-pruritic and erythematous papules, plaques or nodules. The lesions often exhibit a mamillated surface. It is very common to have significant superficial dermal edema leading to a pseudovesicular appearance. Occasionally, the plaques have a central yellowish coloration exhibiting a targetoid appearance. The distribution is often asymmetrical. Nodular lesions involving the lower legs may resemble erythema nodosum, as verified by a small number of patients who presented concurrent erythema nodosum.^5^

The patient did not have the typical manifestations of SS described above. The lesions had an annular pattern, which suggested other differential diagnoses such as erythema multiforme, sarcoidosis and granuloma annulare. Based on the clinical evolution of the patient, laboratory tests and histopathological evaluation, these hypotheses were discarded.

Histopathology revealed a diffuse infiltrate of mononuclear cells in the upper dermis, with many myeloperoxidase-positive cells with histiocytic appearance, corresponding to immature cells of myeloid lineage.^6^ An important differential diagnosis was leukemia cutis, which corresponds to cutaneous infiltration of neoplastic leukocytes.^7^ Both diseases have similar immunoprofiles (immunoreactivity for lysozyme, myeloperoxidase, CD43, CD45 and CD68) and often indistinguishable cytological aspect.^6^ This makes leukemia cutis a major challenge in the differential diagnosis. However, based on clinical manifestations and on the absence of immature myeloid cells in the peripheral blood, HSS is the most appropriate diagnosis for the case.

Histopathologic findings are key to establish a definitive diagnosis of HSS, which cannot be based solely on clinical criteria. The histology of this case emphasizes the fact that neutrophils are not necessarily the predominant cells in SS infiltrates. If HSS is reported, treatment, monitoring and possible associated diseases are the same as the ones described in the classical form of SS. In our opinion, dermatologists should have knowledge of this entity in order not to exclude SS from their diagnosis in the absence of classical histopathological findings. As the patients with HSS are referred as having an unknown risk of developing malignant disorders, they will possibly be better managed with the advancement in molecular research.^8^
